# How parental involvement in youth sports impacts on school adjustment: a dual mediation pathway via sports interest and extracurricular sports participation

**DOI:** 10.3389/fpubh.2025.1621980

**Published:** 2025-07-04

**Authors:** Haoyuan Zheng, Zubing Xiang, Wenli Yang

**Affiliations:** College of Physical Education, Chongqing University, Chongqing, China

**Keywords:** adolescents, school adjustment, parental involvement in youth sports, sports interest, extracurricular sports participation

## Abstract

**Introduction:**

This study aimed to examine whether parental involvement in youth sports influences Chinese adolescents’ school adjustment and to investigate the mediating roles of extracurricular sports participation and sports interest in this relationship.

**Methods:**

Using longitudinal data from the China Education Panel Survey (CEPS) for the 2013–2014 academic year, which included 12,257 junior high school students from China, this study employed a multiple mediation model to analyze the effects of parental involvement in youth sports, sports interest, and extracurricular sports participation on adolescents’ school adjustment.

**Results:**

The findings revealed that: (1) Parental involvement in youth sports had a significant and independent positive effect on adolescents’ school adjustment. (2) Parental involvement in youth sports could also enhance school adjustment by increasing sports interest and promoting extracurricular sports participation, through three distinct pathways: parental involvement in youth sports → extracurricular sports participation → school adjustment; sports interest → extracurricular sports participation → school adjustment; parental involvement in youth sports → sports interest → extracurricular sports participation → school adjustment. These results elucidate the mechanisms through which parental involvement in youth sports, sports interest, and extracurricular sports participation collectively contribute to adolescents’ school adjustment, providing empirical evidence for strategies aimed at improving school adaptation.

**Discussion:**

The study demonstrates that parental involvement in youth sports can directly enhance adolescents’ school adjustment, as well as indirectly influence it through sports interest or extracurricular sports participation individually, or via a multiple mediation of sports interest followed by extracurricular sports participation.

## Introduction

Schools are an important setting for adolescent activities, as adolescents spend most of their time in school (1). Adolescents’ ability to adapt to school life is a key factor in their academic success and overall well-being (2, 3). According to the Seventh National Population Census data released by China’s National Bureau of Statistics in 2021, the floating population in China reached approximately 376 million, accounting for about 27% of the total population, with 125 million being inter-provincial migrants. After experiencing residential relocation, adapting to the social life in their new locations has become a prominent challenge for this mobile population. For migrant children in particular, adapting to collective life in new schools represents a common issue they must directly confront.

The concept of school adjustment was first proposed in Cowen’s AML model in 1973, primarily referring to students’ adaptation to changes in learning environments and academic tasks (4). School adjustment encompasses not only students’ performance in school, but also their feelings or attitudes toward school, as well as their level of participation in school activities (5). It also includes adaptation to school environment and teacher-student/peer relationship adaptation. Positive school adjustment facilitates adolescents’ establishment of positive peer relationships and social skills, enabling them to better cope with academic and life challenges while reducing psychological stress. Furthermore, it correlates with better development in adulthood (6). School adjustment plays a crucial role in adolescents’ healthy development and may significantly influence their future lives (7), whereas school maladjustment serves as a predictor for externalizing problems during adolescence (8). A nationwide longitudinal study in South Korea revealed that school maladjustment was associated with adolescent dropout, depression, substance overuse, digital device addiction, and unhealthy eating habits and these adverse outcomes may persist into adulthood, leading to mental health issues and low productive engagement (9). What’s more, adolescents of different genders exhibit distinct characteristics in school adjustment, necessitating differentiated intervention approaches (10). Therefore, improving school adjustment holds significant research implications for adolescent development.

Extracurricular sports activities serve as a protective factor for adolescent mental health (11), helping teenagers alleviate negative emotions such as anxiety, depression, and frustration (12, 13). Through sports participation, adolescents can establish peer relationships while developing and enhancing social skills and open-mindedness, thereby strengthening their school adjustment capacity (14–16), and promoting positive mental health development (17). A Chinese study revealed that adolescents participating in extracurricular sports activities demonstrated better performance across three dimensions: cooperation, interpersonal communication, and open-mindedness and these findings indicate a significant correlation between extracurricular sports participation and overall enhancement of social and emotional skills (18). Moreover, a study found that adolescents participating in school-based extracurricular physical activities demonstrated better adaptive capacities, reporting improved academic performance, more positive attitudes toward school, and higher educational aspirations (19). The underlying reason may be that interest in extracurricular sports activities typically reflects a stronger “bonding to school” (20). Catalano et al. (21), building upon attachment, control, and social development theories, proposed that strengthening “bonding to school” can cultivate children’s adaptability to change, reduce behaviors detrimental to academic success, and foster achievement within school settings. This enhancement of school adjustment promotes better adolescent development (10). The youth sport experience begins with an interest in participation by children (22). There may be gender differences in adolescents’ interest in extracurricular sports participation. As adolescents grow older, they may exhibit different characteristics in terms of physical, psychological, and social development. Previous studies have shown that adolescent girls are at a higher risk of dropping out of sports earlier than boys (23). There are gender differences in interest toward sports activities, with boys generally showing more interest in extracurricular sports participation than girls (24). Efforts to improve parental support for sport participation may empower girls with greater perceived competence and self-efficacy to capitalize on existing sporting opportunities or seek new opportunities (25).

Family plays an irreplaceable role in children’s socialization process, with parents serving as particularly crucial agents. Brizuela and García-Sellers (26) conceptualize school adjustment as a tripartite process involving dynamic interactions among three key elements: the child themselves, the family unit, and the school environment. A nationally representative Irish cohort study on children confirmed that maternal, paternal, and teacher influences all significantly shape children’s school adjustment (27). Further academic research on parenting styles has revealed that parental involvement plays a significant role in fostering children’s school adjustment and promoting adolescent development (28). Parent–child co-participation in sports activities enhances physical activity interactions, subtly shapes adolescents’ sports values (29), and increases their enjoyment (30). These benefits collectively boost motivation for physical activity, promote youth sports engagement (31), and ultimately improve adolescents’ physical and mental health outcomes (32). A study has shown that parental involvement positively influences adolescents’ academic and athletic performance, and engaging parents in children’s sports activities and organizing youth sports programs represents an effective approach to better facilitate the balance between academic pursuits and physical exercise (33).

With the deepening of research, “parental involvement in youth sports”—specifically the frequency of joint sports participation between adolescents and their fathers or mothers—has garnered widespread attention. In recent decades, youth sports have indeed become both an extension of family life and a component of parental cultivation programs, and this stands in marked contrast to previous eras when most parents were absent from sports fields, as parental engagement in youth sports has increased substantially (34). Through their involvement in youth sports as a parenting practice, parents foster parent–child interactions and fulfill their parental roles. In most cases, adolescents desire their parents’ participation in their sports activities (35). But the reality of parental involvement in youth sports presents considerable complexity (36). Current research provides limited examination of two critical dimensions: the specific pathways through which parental involvement in youth sports fosters school adjustment, and the interrelationships among extracurricular sports participation, parental sports involvement, and sports interest. This gap in the literature has hindered in-depth exploration of the tripartite dynamic between parental factors, sports engagement, and adolescent in the development process of school adjustment.

**Figure 1:**
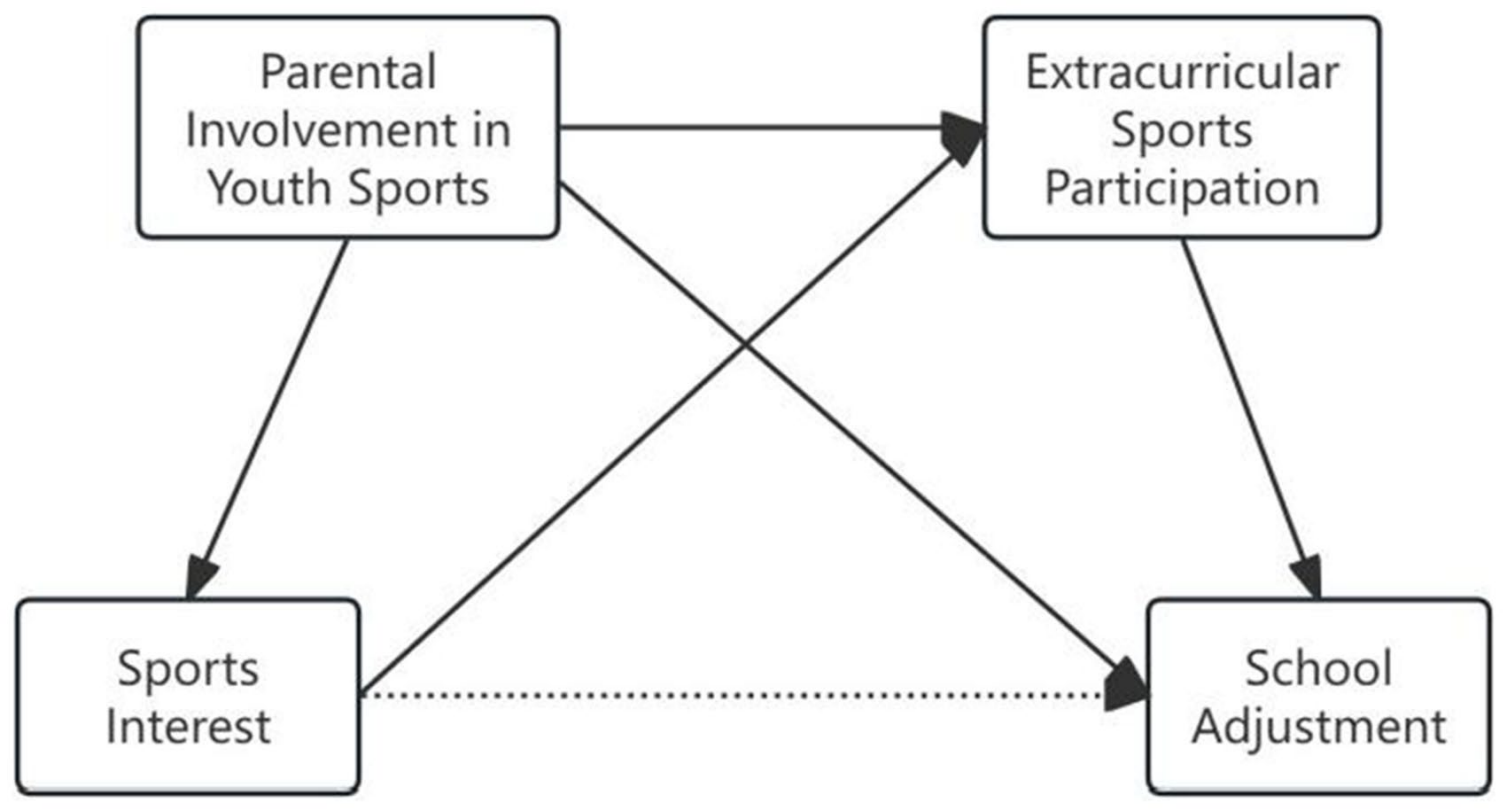
Model of hypothetical relationship. The arrows represent the impact paths.

In summary, school adjustment significantly influences adolescents’ physical and mental health as well as their future development. Extracurricular sports participation, parental involvement in youth sports, and sports interest are intrinsically linked to adolescents’ school adjustment. Utilizing data from the China Education Panel Survey (CEPS), this study aims to further elucidate the impact of parental sports companionship on adolescents’ school adjustment levels, while examining the mediating roles of extracurricular sports participation and sports interest. Accordingly, the study proposes the following hypotheses, and the hypothesized model is illustrated in Figure 1:

*H1*: Parental involvement in youth sports exerts a direct positive effect on adolescents’ school adjustment.

*H2*: Parental involvement in youth sports significantly influences both the duration of adolescents’ physical exercise and their sports interest.

*H3*: Sports interest directly enhances the adolescents extracurricular sports participation.

*H4*: Sports interest mediates the positive relationship between parental involvement in youth sports and adolescents’ physical exercise duration.

*H5*: Extracurricular sports participation serves as a mediator in the positive association between parental involvement in youth sports and adolescents’ school adjustment.

*H6*: A multiple mediation effect exists, where both extracurricular sports participation and sports interest sequentially mediate the positive impact of parental involvement in youth sports on adolescent school adjustment.

## Data variables and methodology

### Data source

This study utilizes data from the baseline survey of the China Education Panel Survey (CEPS) conducted in 2013–2014. The survey was designed and implemented by the National Survey Research Center (NSRC) at Renmin University of China. It is the first large scale, nationally representative tracking survey project in China that starts from the junior high school stage. In 2013, the CEPS adopted a stratifed, multi-stage probability proportional to size (PPS) sampling method. A total of 112 schools and 438 classes were surveyed nationwide, with all students in the selected classes included in the sample. The baseline survey collected data from approximately 20,000 participants. To mitigate the potential impact of outliers, the study applied winsorization to the extracurricular sports participation variable by trimming values above the 99th percentile. After removing responses with missing values and outliers related to the core variables, a final dataset of 12,257 students was retained for empirical analysis.

### Variables

#### Dependent variable

##### School adjustment

The CEPS student questionnaire includes five items assessing adolescents’ school adjustment levels: “Regarding school life, do you agree with the following statements: (1) Most classmates are friendly to me; (2) I find it easy to get along with others; (3) My class has good discipline and atmosphere; (4) I often participate in activities organized by the school or class; (5) I feel close to people at this school.” Response options for each item were: “Strongly disagree” (coded as 1), “Somewhat disagree” (coded as 2), “Somewhat agree” (coded as 3), and “Strongly agree” (coded as 4). The total score ranged from 5 to 20, with higher scores indicating better school adjustment. The scale demonstrated good reliability with a Cronbach’s *α* coefficient of 0.80 (see Table 1).

**Table 1:** Descriptive statistics of the dependent variable responses.

Measurement variable	Observed indicators	Value assignment	Frequency	Percentage
School adjustment	C1: Regarding school life, do you agree with the following statements: my homeroom teacher often praises me	1 “Strongly disagree”	450	3.67%
		2 “Disagree”	938	7.65%
		3 “Agree”	5,177	42.24%
		4 “Strongly agree”	5,692	46.44%
	C2: Regarding school life, do you agree with the following statements: most classmates are friendly to me	1 “Strongly disagree”	537	4.38%
		2 “Disagree”	1,413	11.53%
		3 “Agree”	5,265	42.96%
		4 “Strongly agree”	5,042	41.14%
	C3: Regarding school life, do you agree with the following statements: I find it easy to get along with others	1 “Strongly disagree”	600	4.90%
		2 “Disagree”	1,636	13.35%
		3 “Agree”	4,910	40.06%
		4 “Strongly agree”	5,111	41.70%
	C4: Regarding school life, do you agree with the following statements: the class atmosphere is good	1 “Strongly disagree”	1,549	12.64%
		2 “Disagree”	3,011	24.57%
		3 “Agree”	4,190	34.18%
		4 “Strongly agree”	3,507	28.61%
	C5: Regarding school life, do you agree with the following statements: I often participate in school or class-organized activities	1 “Strongly disagree”	905	7.38%
		2 “Disagree”	2,354	19.21%
		3 “Agree”	5,055	41.24%
		4 “Strongly agree”	3,943	32.17%

#### Independent variable

##### Parental involvement in youth sports

Mediating variables parental involvement in youth sports was measured by the question: “Frequency of doing the following activities with your parents—exercising.” The responses included six options: “1 = never”; “2 = once a year”; “3 = once every 6 months”; “4 = once a month”; “5 = once a week”; “6 = more than once a week.” In this study, the options “once a week” and “more than once a week” were combined and revalued as “at least once a week” (assigned a value of 2), while the other options are combined and revalued as “less than once a week” (assigned a value of 1).

#### Mediating variables

##### Sports interest

In the CEPS survey, students were directly asked about their interest in sports. This variable was measured as a binary (0–1) indicator. “0” indicates no interest in sports, while “1” indicates interest in sports.

##### Extracurricular sports participation

The CEPS 2013–2014 survey directly collected data on respondents’ typical weekly frequency of extracurricular sports participation (days per week) and daily extracurricular sports duration. The average daily extracurricular sports participation time was calculated as: (Weekly extracurricular sports participation days × Daily extracurricular sports participation time) ÷ 7. To make the variable better conform to a normal distribution, this study applied a natural logarithm transformation to the average daily extracurricular sports participation time, thereby creating a normalized continuous variable. To ensure cases with zero average daily extracurricular sports participation time (non extracurricular sports participation) were not excluded from the sample, we added 0.01 to each case’s value before taking the natural logarithm of “average daily extracurricular sports participation time.”

### Data analysis methods

All statistical analyses were performed using Stata 16. The analytical procedures included: descriptive statistics; Spearman’s rank correlation analysis; path analysis; mediation effect testing based on path analysis. Path analysis, a specialized form of structural equation modeling (SEM), was employed to examine the hypothesized causal pathways among observed variables. This analytical approach was particularly suitable for the study because it can simultaneously estimate direct and indirect effects between all variables in the model, more intuitively demonstrate relationships between variables, and provide rigorous empirical data support for verifying the hypothesized model. The significance level was set at *p* < 0.01 for all tests.

## Results

### Descriptive statistics and correlation analysis

The study analyzed data from 12,257 valid participants, including 6,076 male students (coded as 1) and 6,181 female students (coded as 2), with male participants accounting for 49.57% of the total sample. The results showed that adolescents engaged in extracurricular sports for an average of 0.8 h per day, with a standard deviation of 0.879. Regarding parental involvement in youth sports, 59.61% of adolescents reported that their parents participated with them less than once per week, indicating that fewer than half of the adolescents engaged in weekly extracurricular sports with their parents. In terms of sports interest, 60.87% of adolescents showed no interest in sports. For school adjustment measures, the item-level analysis revealed that 15.91% of students either “strongly disagreed” or “somewhat disagreed” with the statement “most classmates are friendly to me”; 26.59% rarely participated in school or class-organized activities; and 18.25% did not feel get along with others at their school. The composite score of the five school adjustment items averaged 15.48 points (score range: 5–20). Notably, 7% of the total sample scored 10 points or below on this measure. Within this low-adjustment subgroup, 72.26% had parents who participated in sports with them less than once weekly, and 68.30% showed no interest in physical exercise.

Spearman correlation analysis (Table 2) demonstrated significant positive correlations (all *p* < 0.01) among school adjustment, parental involvement in youth sports, sports interest, and extracurricular sports participation, supporting subsequent mediation analyses. Notably, while gender showed significant associations with school adjustment, sports interest, and extracurricular sports participation, it was uncorrelated with parental involvement in youth sports. This pattern justifies gender-stratified analyses to examine potential differential effects of parental involvement on school adjustment across sexes.

**Table 2:** The mean, standard deviation and the correlation coefficient.

Variable	M	SD	School adjustment	Parental involvement in youth sports	Sports interest	Extracurricular sports participation	Gender
School adjustment	15.478	3.249	1.000				
Parental involvement in youth sports	1.404	0.491	0.234^*^	1.000			
Sports interest	0.391	0.488	0.087^*^	0.056^*^	1.000		
Extracurricular sports (ln)	−1.252	2.055	0.169^*^	0.189^*^	0.253^*^	1.000	
Gender	1.504	0.500	0.064^*^	−0.003	−0.260^*^	−0.118^*^	1.000

^*^Indicates statistical significance at *p* < 0.01 level.

### Hypothesis testing

The study conducted path analysis with maximum likelihood estimation to examine a multiple mediation model where parental involvement in youth sports, sports interest and extracurricular sports participation served as the independent variable, and school adjustment as the dependent variable.

The results demonstrated that: (1) Parental involvement in youth sports exerted significant positive direct effects on school adjustment, sports interest, and extracurricular sports participation; (2) Extracurricular sports participation showed a significant positive direct effect on school adjustment; (3) Sports interest significantly and positively predicted extracurricular sports participation.

**Figure 2:**
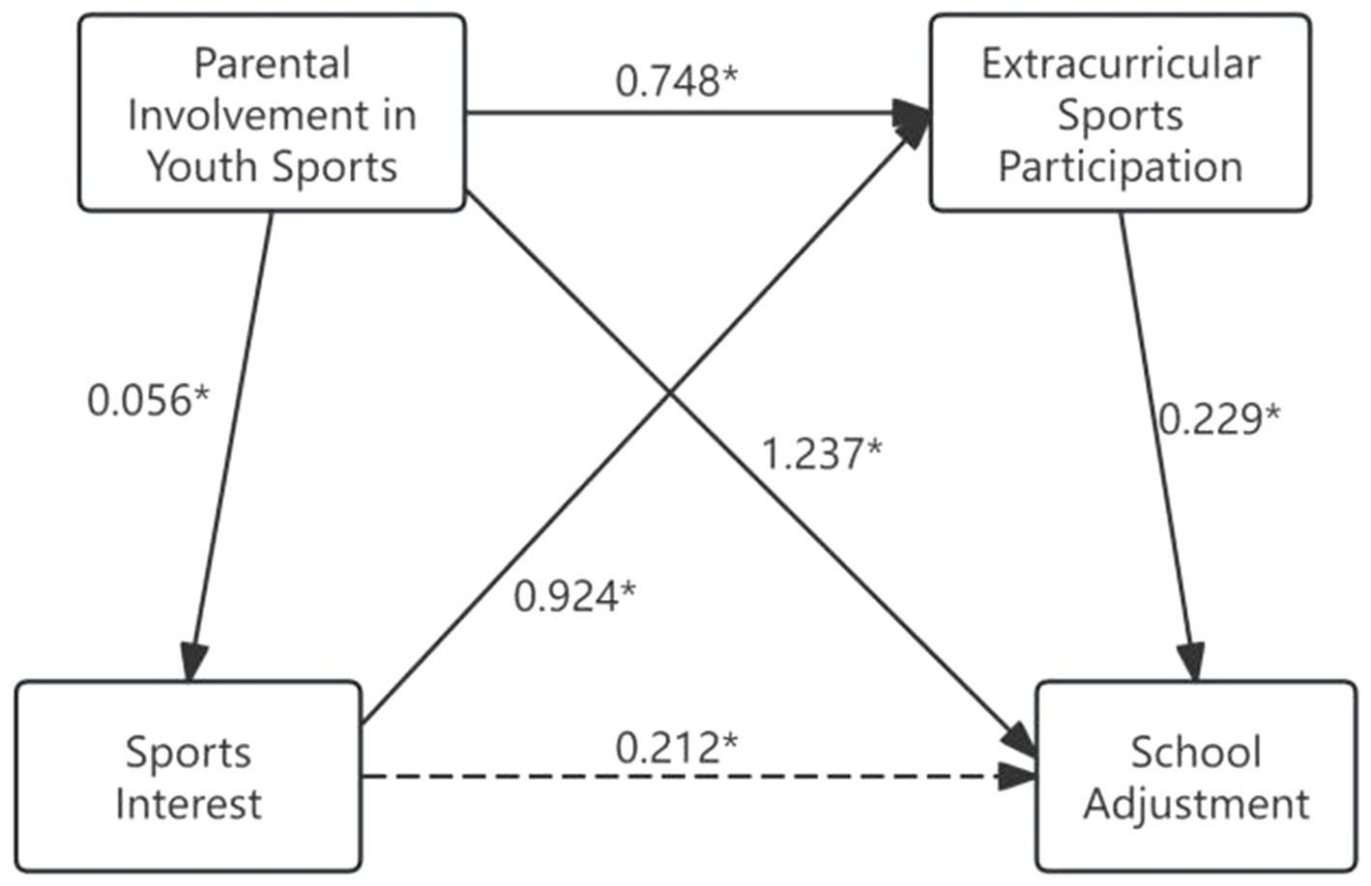
Path diagram for testing mediating effect. The arrows represent the impact paths. ^*^Indicates statistical significance at *p* < 0.01 level. Solid lines represent direct effects, while dashed lines denote indirect effects.

The mediation analysis results (Table 3) revealed significant indirect effects of sports interest and extracurricular sports participation in the relationship between parental involvement in youth sports and school adjustment. As illustrated in Figure 2, these mediating effects operated through three specific pathways: Path 1: parental involvement in youth sports → extracurricular sports participation → school adjustment. In this pathway, parental involvement in youth sports directly increased extracurricular sports participation which subsequently enhanced school adjustment levels; Path 2: sports interest → extracurricular sports participation → school adjustment. This pathway demonstrated that adolescents’ sports interest enhanced their school adjustment through increased extracurricular sports participation; Path 3: parental involvement in youth sports → sports interest → extracurricular sports participation → school adjustment. In this pathway, parental involvement in youth sports improved school adjustment by first enhancing adolescents’ sports interest, which subsequently increases their extracurricular sports participation, ultimately leading to better school adjustment. The analysis revealed that, compared to adolescents’ own extracurricular sports participation, parental involvement in youth sports demonstrated a higher coefficient of influence on their school adjustment. This finding further validated the model hypothesis and highlighted the critical role of parental involvement in youth sports in shaping adolescents’ school adjustment.

**Table 3:** Mediating effect analysis.

Results	Direct effect	Indirect effect	Total effect
Parental involvement in youth sports → school adjustment	1.237^*^	0.183^*^	1.421^*^
Sports interest → school adjustment		0.212^*^	0.212^*^
Extracurricular sports participation → school adjustment	0.229^*^		0.229^*^
Sports interest → extracurricular sports participation	0.924^*^		0.924^*^
Parental involvement in youth sports → extracurricular sports participation	0.748^*^	0.051^*^	0.799^*^
Parental involvement in youth sports → sports interest	0.056^*^		0.056^*^

^*^Indicates statistical significance at *p* < 0.01 level.

### Gender difference testing in the mediation model

Consistent with prior research demonstrating significant gender differences in adolescents’ extracurricular sports participation, sports interest, and school adjustment, this study conducted gender-stratified analyses to examine potential variations in the mediation effects. Using the same analytical approach, this study separately tested the mediating roles of sports interest and extracurricular sports participation in the relationship between parental involvement in youth sports and school adjustment for male and female adolescents.

As shown in Table 4, parental involvement in youth sports exerted significant positive direct effects on male adolescents’ sports interest, extracurricular sports participation, and school adjustment. Both extracurricular sports participation and school adjustment demonstrated partial mediating effects, with evidence of sequential mediation (parental involvement in youth sports → school adjustment through multiple pathways). For female adolescents, parental involvement in youth sports demonstrated consistent directional effects on school adjustment, sports interest, and extracurricular sports participation as observed in males. Notably, the direct and indirect effects of parental involvement in youth sports on school adaptation were higher for girls than for boys. Similarly, the direct effect and total effect of parental involvement in youth sports on extracurricular sports participation were also greater for girls. These findings suggest that, compared to boys, parental involvement in youth sports plays a more influential role in increasing extracurricular sports participation and shaping girls’ school adjustment.

**Table 4:** Gender-stratified analysis of mediation effects.

Results	Direct effect		Indirect effect		Total effect	
	Male	Female	Male	Female	Male	Female
Parental involvement in youth sports → school adjustment	1.145^*^	1.317^*^	0.180^*^	0.202^*^	1.324^*^	1.518^*^
Sports interest → school adjustment			0.262^*^	0.164^*^	0.262^*^	0.164^*^
Extracurricular sports participation → school adjustment	0.259^*^	0.224^*^			0.259^*^	0.224^*^
Sports interest → extracurricular sports participation	1.013^*^	0.734^*^			1.013^*^	0.734^*^
Parental involvement in youth sports → extracurricular sports participation	0.630^*^	0.867^*^	0.066^*^	0.033^*^	0.695^*^	0.900^*^
Parental involvement in youth sports → sports interest	0.065^*^	0.045^*^			0.065^*^	0.045^*^

^*^Indicates statistical significance at *p* < 0.01 level.

## Discussion

Drawing on data from the China Education Panel Survey (CEPS), this study tested the proposed hypotheses and examined the relationship between parental involvement in youth sports and school adjustment. The findings indicated that parental involvement in youth sports significantly and positively predicted adolescents’ interest in sports and their extracurricular sport participation. This suggested that parental involvement in youth sports facilitated both the cultivation and stimulation of adolescents’ sports interest, as well as their extracurricular sport participation. Parental involvement in youth sports can indirectly influence school adjustment through a multiple mediation pathway by first enhancing adolescents’ sports interest, which subsequently increases their extracurricular sports participation. Although there is insufficient evidence to suggest that sports interest directly influences adolescents’ school adjustment, fostering and stimulating sports interest can indirectly affect school adjustment by increasing extracurricular sports participation. The mechanism through which parental involvement in youth sports influenced adolescents’ school adjustment did not differ by gender and aligned with the pattern observed in the main effects; however, girls appeared to be more susceptible to the influence of parental involvement. Furthermore, the study also revealed that the frequency of parental involvement in youth sports could influence school adjustment by affecting adolescents’ sports interest and extracurricular sports participation. Thus, increasing extracurricular sports participation can, to some extent, mitigate the negative impact of the lack of parental involvement in youth sports on school adjustment.

This study revealed the multiple mediating roles of sports interest and extracurricular sports participation in the relationship between parental involvement in youth sports and school adjustment. The findings reaffirmed the positive influence of active parental involvement in youth sports on adolescents’ school adjustment and highlighted that such involvement can enhance school adjustment by fostering sports interest, which in turn promoted greater participation in extracurricular sports. This study further underscored the importance of family sports culture, as previous research has identified the family sports environment as one of the most critical factors influencing adolescents’ regular participation in physical activity (37). If parents never engage in physical exercise themselves, their children are more likely to be reluctant to participate in physical activity (38). As previous studies have indicated, family cultures were the chief factor underpinning individuals’ propensities (39); as one of the most influential factors affecting sports participation, family sports culture plays a critical role in promoting adolescents’ sustained engagement in physical activity (40). Existing research has shown that, whether through sports or other forms of engagement, parents’ active involvement and companionship in adolescents’ lives significantly enhance their level of school adjustment (28). It is important to note that excessive parental involvement can create pressure for children, who prefer parental involvement characterized by praise and understanding (36). The current frequency of Chinese adolescents participating in extracurricular sport with their parents remains concerning, reflecting the insufficient role of families in promoting adolescent extracurricular sport participation. This is particularly evident among parents of lower socioeconomic status, who are generally less willing to engage in extracurricular sports with their children (41). The reason for this is that lower socioeconomic status may limit opportunities for extracurricular sports due to a lack of transportation and financial resources (42). In addition, the current lack of popular sports skills may also contribute to some parents’ inability to effectively participate in their adolescents’ extracurricular sports. Therefore, the government should pay more attention to families with low socioeconomic status, helping them to overcome their deficiencies in both physical abilities and sports consumption capacity. It is necessary to utilize schools and communities as platforms to make full use of available sports resources, thereby supporting parents in their involvement in youth sports. This study draws on and references relevant findings from previous research, employing a quantitative research method to explore the relationship between parental involvement in youth sports, sports interest, extracurricular sports participation, and school adjustment. As an active intervention for both physical and mental health, extracurricular sport participation has gained widespread recognition among parents globally, and accompanying children in sports activities is becoming a common phenomenon. This study addresses the current theoretical gaps and lack of empirical evidence in research on the relationship between parental involvement in youth sports and adolescents’ school adjustment. By employing a multiple mediation model, the study verifies the positive impact of parental involvement in youth sports on adolescents’ sports interest, extracurricular sports participation, and school adjustment.

At the practical level, this study offers actionable guidance on parent–child extracurricular sport participation for ordinary families and proposes targeted strategies to improve school adjustment among special groups such as left-behind adolescents. At the theoretical level, it preliminarily constructs an analytical framework for understanding the mechanisms through which parental involvement in youth sports influences school adjustment, thereby laying the groundwork for future research.

### Limitations

This study was a cross-sectional study that, based on relevant research strategies, explored predictive outcomes and provided empirical support for understanding the causal relationships between variables. Future research should adopt longitudinal tracking or experimental interventions to investigate causal relationships among the variables. As this study utilized publicly available data to validate the research hypotheses, the selection of measurement schemes for core variables was somewhat passive. Moreover, it should be noted that the current study utilizes data from the CEPS 2013–2014 survey, which was collected approximately a decade ago. Given the potential shifts in cognitive level and behavioral patterns over time, there may be meaningful differences between the survey context and contemporary circumstances that warrant consideration when interpreting these findings. Therefore, the extent to which the relationships between these variables accurately reflected real-world conditions still warrants further investigation. Additionally, this study only considered extracurricular sports participation and sports interest as mediators between parental involvement in youth sports and school adjustment. However, other potential mediating variables, such as self-awareness, psychological resilience, and interpersonal skills, may also play a role and deserve further exploration. Future research could employ a combination of experimental, observational, survey, measurement, and longitudinal approaches for mutual validation. Leveraging advanced scientific technologies, future studies should delve deeper into the impact of variables such as different sports activities and exercise intensity. This approach would enhance the ecological validity of the research, assist in providing more support pathways for adolescents, increase their interest in extracurricular sports activities, and ultimately improve school adjustment levels.

## Data Availability

The original contributions presented in the study are included in the article/supplementary material, further inquiries can be directed to the corresponding author.
